# Impact of COVID-19 national lockdown on asthma exacerbations: interrupted time-series analysis of English primary care data

**DOI:** 10.1136/thoraxjnl-2020-216512

**Published:** 2021-03-29

**Authors:** Syed A Shah, Jennifer K Quint, Bright I Nwaru, Aziz Sheikh

**Affiliations:** 1 Asthma UK Centre for Applied Research, Centre for Medical Informatics, Usher Institute, The University of Edinburgh, Edinburgh, UK; 2 National Heart & Lung Institute, Imperial College London, London, UK; 3 Krefting Research Centre, The University of Edinburgh, Gothenburg, Sweden

**Keywords:** asthma, asthma epidemiology, COVID-19

## Abstract

**Background:**

The impact of COVID-19 and ensuing national lockdown on asthma exacerbations is unclear.

**Methods:**

We conducted an interrupted time-series (lockdown on 23 March 2020 as point of interruption) analysis in asthma cohort identified using a validated algorithm from a national-level primary care database, the Optimum Patient Care Database. We derived asthma exacerbation rates for every week and compared exacerbation rates in the period: January to August 2020 with a pre-COVID-19 period and January to August 2016–2019. Exacerbations were defined as asthma-related hospital attendance/admission (including accident and emergency visit), or an acute course of oral corticosteroids with evidence of respiratory review, as recorded in primary care. We used a generalised least squares modelling approach and stratified the analyses by age, sex, English region and healthcare setting.

**Results:**

From a database of 9 949 387 patients, there were 100 165 patients with asthma who experienced at least one exacerbation during 2016–2020. Of 278 996 exacerbation episodes, 49 938 (17.9%) required hospital visit. Comparing pre-lockdown to post-lockdown period, we observed a statistically significant reduction in the level (−0.196 episodes per person-year; p<0.001; almost 20 episodes for every 100 patients with asthma per year) of exacerbation rates across all patients. The reductions in level in stratified analyses were: 0.005–0.244 (healthcare setting, only those without hospital attendance/admission were significant), 0.210–0.277 (sex), 0.159–0.367 (age), 0.068–0.590 (region).

**Conclusions:**

There has been a significant reduction in attendance to primary care for asthma exacerbations during the pandemic. This reduction was observed in all age groups, both sexes and across most regions in England.

**Key messages**

**What is the key question?**

- What is the impact of COVID-19 and the ensuing national lockdown on healthcare attendance for asthma exacerbations?

**What is the bottom line?**

- Our national-level, interrupted-time series study following a cohort of 100 165 patients with asthma across England showed that there was a substantial reduction in overall primary care-recorded exacerbation rates for both men and women, in all age groups and across most regions in England. This reduction was observed in exacerbations managed in primary care that did not require a hospital visit. Our study found no significant change in more serious exacerbations that required hospital attendances and/or admission.

**Why read on?**

- Existing evidence suggests that having a comorbidity such as asthma is a risk factor of COVID-19-related hospitalisation and possibly death. However, the impact of the pandemic and the ensuing national lockdown on asthma exacerbations is not clear. This study provides national-level insights about the impact of national lockdown measures on healthcare attendance for asthma exacerbations.

## Introduction

Early studies from Wuhan City (China), where COVID-19 caused by SARS-CoV-2 was first identified in December 2019, suggested that COVID-19 can lead to severe pneumonia and life-threatening acute respiratory distress syndrome.^1^ These initial investigations also demonstrated that people with underlying health conditions, including respiratory illnesses, are at higher risk of severe disease and death due to COVID-19.^2^ These findings were further corroborated by studies in the USA,^3 4^ UK^5 6^ and other European countries.^7^

Asthma has been identified as a possible risk factor for COVID-19-associated hospitalisation and death.^3 6^ A significant proportion of asthma-related healthcare resource utilisation and cost is associated with asthma exacerbations.^8^ Overall, asthma exacerbations represent a huge socioeconomic burden both in the UK and across the world. In the UK alone, there are at least 6.3 million primary care consultations, 93 000 hospital episodes and 1400 deaths attributed to asthma every year.^9^

Since the majority of asthma exacerbations are associated with respiratory viral illnesses,^10^ there was initial concern that COVID-19 may lead to increased asthma exacerbations.^11^ However, the evidence to date is not clear. A study from Wuhan reported that only 0.9% patient among the 269 severe cases investigated had asthma.^12^ Another study from Paris (France) reported that patients with asthma were not over-represented among the 768 hospitalised patients with COVID-19.^13^ However, some studies have suggested that the percentage of patients who have asthma among patients with COVID-19 who are hospitalised is higher than asthma prevalence.^14 15^ In the largest population-based risk prediction modelling study to date, asthma has been found to be an independent risk factor for COVID-19 hospitalisations, but not death.^16^

The WHO declared COVID-19 a pandemic on 11 March 2020,^17^ which triggered unprecedented nationally enforced social distancing measures by governments across the globe. These measures included widespread lockdowns of whole societies, including school closures and movement restrictions^18^ that have had numerous negative health, social and economic effects.^19–22^ These lockdowns have however had the beneficial effects of leading to improvements in air quality^23–25^ and a likely reduction in circulating respiratory viruses (especially in children and young people). It is also possible that the earlier concerns about increased risk of severe illness and death from COVID-19 may have led to improved self-management and shielding among patients with asthma. Consequently, we hypothesised that the UK’s national lockdown, which began on 23 March 2020, lead to a reduction in asthma exacerbations in England. We tested our hypothesis by following a large cohort of patients with asthma using a national-level primary care database.

## Methods

### Data source and setting

We used the Optimum Patient Care Database (OPCRD), a live and growing database of de-identified, longitudinal electronic medical records populated by a network of primary care practices from across the UK. OPCRD has been used to conduct epidemiological, pharmaceutical and clinical studies (https://opcrd.co.uk/).^26 27^ At the time of data access, OPCRD consisted of almost 10 million patients from 792 practices. We used data from the 670 practices in England (the remaining practices were from other nations of the UK). In addition to demographic information, the OPCRD dataset contains diagnoses, symptoms, treatment and prescription data encoded with Read codes, a comprehensive system of clinical concepts classification system that has been used in primary care practices across the UK for about three decades.^28^ For this study, we were provided with a secure access to the Microsoft SQL database through a secure remote access.

### Study design and population

We identified a cohort of patients with clinician diagnosed and recorded asthma who were then followed over time to assess if and when they experienced asthma exacerbations. The cohort of patients with asthma was identified with a previously validated algorithm^29^ over the period 1 January 2010 to 31 December 2015. This algorithm comprised of 121 specific asthma codes that have previously been shown to identify patients with asthma from UK primary care records with high accuracy.^29^ The cohort of patients with asthma was then followed from 1 January 2016 to 16 August 2020; and the outcome measure, asthma exacerbations, was determined for every patient for each week. Using this cohort of patients, we designed an interrupted time-series study with control. The follow-up period was divided into a COVID-19 year (the ‘intervention’ period) and pre-COVID-19 years (the ‘control’ period). The single point intervention was the week corresponding to imposition of lockdown (23 March 2020); the control was the mean weekly exacerbation rate (mean of 4 years, 2016–2019 inclusive for January–August), and the intervention period was the weekly exacerbation rate from January to August 2020.

### Ascertainment of outcome

Based on the American Thoracic Society/European Respiratory Society Task Force definition^30^ and previously validated in OPCRD,^31^ an asthma exacerbation in a given assessment period was defined as the presence of either one of the following: an asthma-related accidental and emergency (A&E) department attendance, an asthma-related hospital admission or an acute course of oral corticosteroids (OCS) with evidence of respiratory review within 2 weeks of OCS prescription. In addition to Read codes associated with asthma-associated hospital attendance or admission, we identified additional non-asthma-specific hospitalisation codes and considered a patient to have experienced a hospital-associated asthma exacerbation if a patient had any of the 121 specific asthma codes (previously used to identify patients with asthma^29^) on the same day when they had any hospitalisation code in their records. Online supplemental appendix 1 provides a list of the Read codes used to ascertain the outcome.

10.1136/thoraxjnl-2020-216512.supp1 Supplementary data

### Data analysis

For every assessment period (defined as a week), we determined the total number of exacerbations, normalised this number with the total number of patients in the study in every period and then converted it into an exacerbation rate (total number of exacerbation episodes per patient-year). Once the exacerbation rate was derived for every week from January 2016 to August 2020, the data were split into a COVID-19 year and pre-COVID-19 years. In 2020, the pre-lockdown period corresponded to weeks 1–12 (January–March), and the post-lockdown period corresponded to the period starting from week 13 (week starting 23 March). Every year in the follow-up (2016–2019) was divided into two periods (weeks 1–12 and weeks 13–32) to allow a comparison of those years with the year 2020 when the national lockdown was imposed. Data were then analysed using an interrupted time-series design with control. First, ordinary least squares regression (OLS) analysis was applied with eight coefficients to be determined. The eight coefficients were: an intercept term and existing trend; existing level and trend difference; post-intervention level and trend; and level change and trend change difference. The OLS model was then tested for the presence of ‘autoregression’ and ‘regression’ type relationships in the data (autocorrelation type relationships are expected in data with seasonality pattern) with autocorrelation and partial autocorrelation plots. This step also helped to determine the order of moving average and/or autoregression relationship in the data. Subsequently, a generalised least squares model was fitted to the data incorporating both the autoregression and moving average relationship in the data. The final fitted model was then used to extract the absolute and relative changes in the outcome of interest. In our study, the outcomes of interest were overall exacerbations, exacerbations that were resolved within primary care without any hospital visit, exacerbations that required a hospital visit, and overall exacerbation stratified by age groups, sex and region. Interrupted time-series analysis allows us to measure two potential changes that could occur because of the intervention: a change in level and a change in trend. Change in level corresponds to the sudden change in the exacerbation rate immediately after the intervention. A change in trend corresponds to difference in the trend (slow change of exacerbation rate over time) between pre-intervention and post-intervention periods.

All analyses were undertaken in R Studio (V.1.2.5033) using R (V.3.6.2). The tidyverse packages^32^ were used for data manipulation (dplyr), date manipulations (lubridate) and data visualisations (ggplot2). The nlme package^33^ was used for building the generalised least squares model. The final figures illustrating the results of the interrupted time series were plotted with Matlab (R2018a, Mathworks, USA).^34^

### Study reporting

This study is reported following the recommendations of Reporting of studies Conducted using Observational Routinely-collected Data.^35^

## Results

### Baseline characteristics of the study population

**Figure 1:**
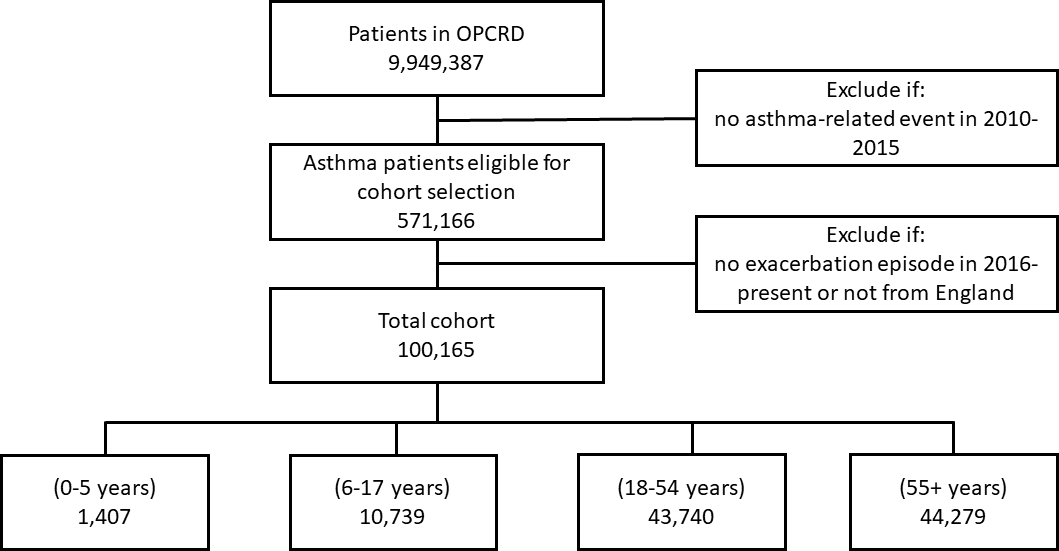
Overall flow diagram of patients in the study. OPCRD, Optimum Patient Care Database.

Out of the 571 166 patients with asthma identified (5.7% of the total population in OPCRD), 100 165 patients (17.5% of those identified with asthma) had at least one exacerbation during the follow-up period (January 2016–August 2020). The total follow-up time in the study was 416 639 patient-years (mean follow-up of 4.16 patient-years). Figure 1 provides a flow diagram of the patients in the study. There were more women (60 360; 60.1%) than men (39 673; 39.6%) with a small number with missing sex information (132; 0.1%). Most patients in the study were those aged 18–54 (43 740; 43.7%) and ≥55 years (44 279; 44.2%). The number of young patients with asthma was relatively low: 0–5 years (1407; 1.4%), 5–18 years (10 739; 10.7%). A large proportion of the patients in the cohort were from East England (21 002; 21%), South East (20 445; 20.4%) and Yorkshire and the Humber (17 240; 17.2%). The remaining patients were from East Midlands (4483; 4.5%), London (3302; 3.3%), North East (4985; 5.0%), North West (12 456; 12.4%), South West (14 378; 14.4%) and West Midlands (1874; 1.9%).

### Exacerbation pattern during follow-up

**Figure 2:**
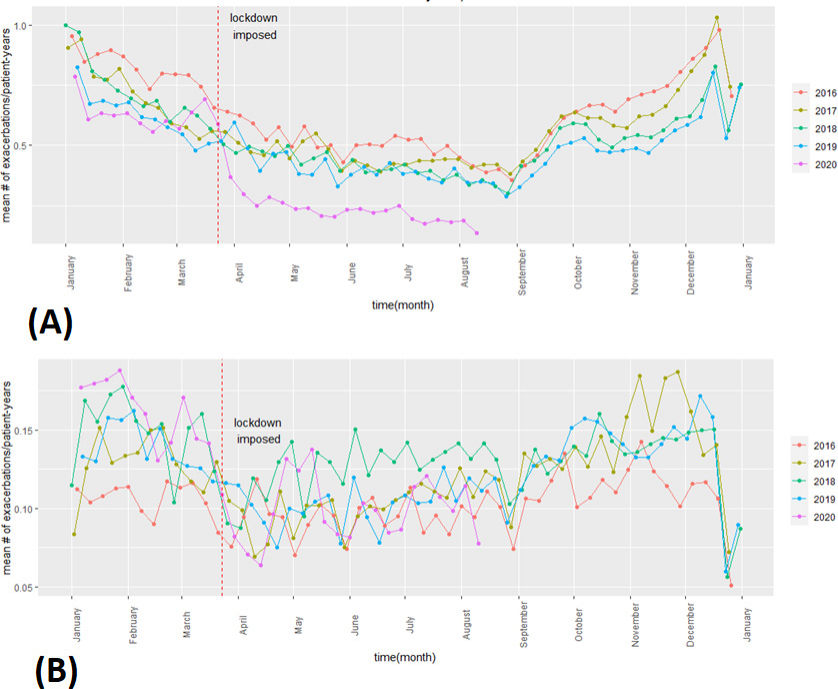
Mean exacerbation rate for every week, from January 2016 to August 2020 across England stratified by exacerbation type: (A) primary care-based exacerbations only and (B) hospital-based exacerbations only.

Figure 2 provides the mean exacerbation rate of the patient cohort for every week during the follow-up period (January 2016–August 2020) categorised by whether patients were managed by general practitioners (GPs) only (termed primary care based in the figure) or whether patients eventually attended hospital (termed hospital based in the figure, which included both A&E attendance and hospital admission). The figure illustrates the seasonality pattern in both categories: the exacerbation rate gradually decreased from January onwards until summer, and it then gradually increased again from August/September until December/January. The sharp decrease during the last week of December corresponded to restricted opening times of primary care practices. Online supplemental appendix 2 provides the mean exacerbation rate of all patients in the cohort during follow-up categorised by sex (online supplemental figure S1), age (online supplemental figure S2) and region (online supplemental figure S3).

10.1136/thoraxjnl-2020-216512.supp2 Supplementary data

During follow-up, there were a total of 278 996 exacerbation episodes experienced by the 100 165 patients. Of these, 229 058 (82.1%) were managed exclusively in primary care without requiring any hospital visit, and the remaining 49 938 (17.9%) required a hospital visit. Table 1 provides the mean exacerbation rate for the patients in the cohort in two distinct periods: weeks 1–12 and 13–32. There was a significant drop in the mean exacerbation rate for all years during the follow-up when comparing weeks 1–12 (corresponding to January–March) with weeks 13–32 (corresponding to April–August).

**Table 1:** Total number of patients and exacerbation rate over time (mean rates for each period given, weeks refer to ISO weeks)

		Exacerbation rate (number of episodes/person-year) mean (95% CI)		
Period	Number of patients in cohort	Resolved within primary care only	Required hospital visit	Overall
2016 (weeks 1–12)	100 165	0.82 (0.76 to 0.87)	0.11 (0.10 to 0.11)	0.92 (0.87 to 0.98)
2016 (weeks 13–32)	99 813	0.52 (0.49 to 0.55)	0.10 (0.09 to 0.10)	0.61 (0.58 to 0.64)
2017 (weeks 1–12)	98 264	0.71 (0.62 to 0.80)	0.13 (0.12 to 0.14)	0.84 (0.75 to 0.93)
2017 (weeks 13–32)	97 689	0.46 (0.43 to 0.48)	0.10 (0.09 to 0.11)	0.56 (0.53 to 0.58)
2018 (weeks 1–12)	96 012	0.73 (0.64 to 0.82)	0.15 (0.13 to 0.16)	0.88 (0.79 to 0.97)
2018 (weeks 13–32)	95 410	0.43 (0.40 to 0.45)	0.13 (0.12 to 0.13)	0.55 (0.53 to 0.57)
2019 (weeks 1–12)	91 439	0.62 (0.55 to 0.68)	0.14 (0.13 to 0.15)	0.75 (0.68 to 0.82)
2019 (weeks 13–32)	88 976	0.41 (0.38 to 0.44)	0.10 (0.10 to 0.11)	0.51 (0.48 to 0.54)
2020 (weeks 1–12)	77 258	0.63 (0.59 to 0.67)	0.16 (0.14 to 0.17)	0.78 (0.74 to 0.83)
2020 (weeks 13–32)	67 995	0.23 (0.20 to 0.25)	0.10 (0.09 to 0.11)	0.33 (0.30 to 0.35)

Total number of exacerbation episodes: 278 996; exacerbation episodes managed exclusively by a general practitioner: 229 058 (82.1%); exacerbation episodes requiring hospital visit: 49 938 (17.9%).

### Interrupted time-series analyses

Table 2 provides results of the interrupted time-series analyses (the rates in this table express the number of exacerbations per person-year). The intercept (akin to a mean value when trend and change due to intervention is separately accounted for) in the table shows that: there were more exacerbations that did not require hospital attendance/admission and were resolved within primary care compared with those that required hospital attendance/admission (0.913 vs 0.134); overall, women were slightly more likely to experience exacerbations than men (1.090 vs 0.976); and the exacerbation rate was highest in the ≥55 age group (1.305) followed by 18–54 (0.919) and 0–5 (0.839) and lowest in 5–17 (0.618). Based on regions in England (as represented in OPCRD), West Midlands had the highest rate of exacerbation (1.383) followed by the North East (1.205), while London had the lowest rate of exacerbation (0.639).

**Table 2:** Intercept, residual SE, and change in level and trend after lockdown was imposed with corresponding p values (compared with average of last 4 years, the control group)

Cohort	Intercept	Change in level after intervention	Change in trend after intervention	Residual SE
All patients, n=100 165	0.833*	−**0.196** (**0.0077**)	0.029 (0.0536)	0.082
Stratification by healthcare setting				
Resolved within primary care	0.913*	−0.244*	−**0.022** (**0.0001**)	0.032
Hospital	0.134*	−0.005 (0.7894)	0.004 (0.1149)	0.014
Stratification of population cohort by sex†				
Men, n=39 673	0.976*	−0.210*	−**0.009** (**0.0068**)	0.035
Women, n=60 360	1.090*	−0.277*	−**0.024** (**0.0015**)	0.042
Stratification of population cohort by age (years)				
0–5, n=1407	0.839*	−**0.367** (**0.0013**)	−0.009 (0.5175)	0.127
5–17, n=10 739	0.618*	−**0.159** (**0.0328**)	−0.005 (0.6351)	0.061
18–54, n=43 740	0.919*	−**0.238** (**0.0010**)	−**0.037** (**0.0004**)	0.059
≥55, n=44 279	1.305*	−0.241*	0.002 (0.7942)	0.041
Stratification of population cohort by region				
East England, n=21 002	1.067*	−0.283*	−**0.014** (**0.0307**)	0.048
East Midlands, n=4483	0.787*	−0.371*	−**0.037** (**0.0003**)	0.083
London, n=3302	0.639*	−0.261 (0.0702)	−0.004 (0.8451)	0.133
North East, n=4985	1.205*	−0.068 (0.6176)	−0.025 (0.1845)	0.108
North West, n=12 456	1.186*	−**0.258** (**0.0147**)	−**0.060** (**0.0001**)	0.089
South East, n=20 445	1.030*	−0.261*	0.000 (0.9582)	0.051
South West, n=14 378	1.049*	−0.275*	−**0.010** (**0.0028**)	0.050
West Midlands, n=1874	1.383*	−**0.590** (**0.0031**)	−0.146*	0.191
Yorkshire and the Humber, n=17 240	0.989*	−**0.132** (**0.0235**)	−0.011 (0.1555)	0.048

*P<0.0001.

†A total of 132 patients did not have sex information in the database.

Overall, there was a statistically significant change in level (−0.196; p=0.008) of exacerbation rate. The change in trend was, however, not significant (0.029; p=0.054). When exacerbations were categorised into whether they were managed exclusively in primary care with no need of hospital attendance and/or admission, or whether they required hospital visit, we only found a statistically significant difference in level (−0.244; p=0.000) and trend (−0.022; p=0.000) in the former case. Figure 2 shows mean exacerbation rate for every week, from January 2016 to August 2020 across England stratified by exacerbation type: (a) primary care-based exacerbations only and (b) hospital-based exacerbations only.

**Figure 3:**
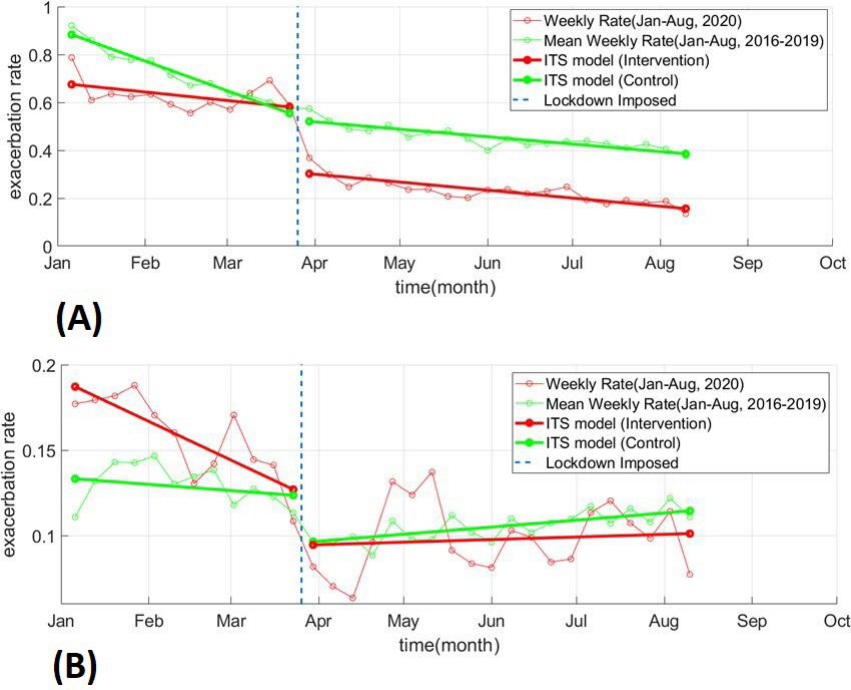
Interrupted time-series (ITS) model fitted to yearly exacerbation rate for every week from January to August for control group (mean rate from 2016 to 2019) and the intervention group (2020): (A) exacerbations related to primary care consultations only and (B) hospital-based exacerbations only.

Figure 3 illustrates the result of the interrupted time-series analyses for both of these categories. Table 2 provides results of the interrupted time-series analyses when the cohort was stratified by sex, age and region. Stratified by sex, there is a significant reduction in trend and level for both men and women. Stratified by age, there was a significant drop in all groups with the most notable decrease in the youngest age group (aged 5–10 years in 2020). When stratified by region, there was a significant drop in level across all regions except London and the North East.

## Discussion

We found a significant overall decrease in asthma exacerbations in England during the COVID-19 pandemic after the lockdown was imposed on 23 March 2020. The decrease was statistically significant in all age groups, in both men and women, and almost across all regions in England. The primary care dataset allowed us to assess whether a given exacerbation episode was resolved within primary care or whether it required a hospital visit. Throughout follow-up (January 2016–August 2020), most asthma exacerbations were resolved within primary care without requiring any hospital visit. We found a statistically significant drop in exacerbation episodes that did not require hospital visit, after the lockdown was imposed. There was no significant decrease in exacerbation episodes that required hospital visit. Overall, our findings indicate that there was a substantial decrease in asthma exacerbations that did not require hospital visit (likely mild cases), but insufficient evidence to suggest any impact on those (presumably more severe) cases of asthma exacerbations that required a hospital visit.

To our knowledge, this is the first national-level study that assessed the impact of the COVID-19 pandemic on attendance to primary or secondary care for asthma exacerbations. The key strengths of this study are a long follow-up (January 2016–August 2020), and the use of validated algorithms to identify patients with asthma and asthma exacerbations in a national primary care database from multiple centres covering a wide geographical area. This study used all English data in the OPCRD that were collected as part of routine clinical care pathway thereby minimising both selection and information bias often associated with observational studies.^36^

There are some limitations to our study. We only had access to primary care records and while primary care will be the first point of contact for any patient with asthma, it is likely that some patients might have attended A&E department in hospital without referral. Our study may miss any such episodes, particularly if there is no subsequent primary care follow-up by the patient or if there is no communication between primary and secondary care. While it is not possible to ascertain the number of such episodes due to absence of any linked data (to link primary care records with A&E), we believe that such occurrences will be rare since patients with chronic condition (such as asthma) are likely to make contact with primary care for follow-up and/or medication. In addition, a discharge letter is typically sent to primary care following any hospital discharge which then gets added to a patient’s primary care record. Second, while interrupted time-series analyses quantitatively show the extent of difference observed after an intervention, it cannot ascertain any causal relationships. Lastly, we have assumed that the approach of GPs in using the Read codes to manage patients with asthma has not changed during the pandemic.

It is important to highlight that our analysis included all patients with asthma who had at least one exacerbation during the follow-up period. While we could include all patients in the analysis, it will not change the results of the interrupted time-series analysis. This is because these excluded patients had zero exacerbation episodes and therefore the relative change in exacerbation rate from pre-COVID-19 years to COVID-19 years will not change (only the absolute value of the exacerbation rate, and hence the intercept value in the interrupted time-series analysis will change across the whole time series).

Since viral respiratory infections are the main trigger of asthma exacerbations,^10^ there were concerns at the start of the pandemic that COVID-19 may have led to increase in asthma exacerbations and that COVID-19 outcomes would be poorer in those with asthma. Several studies, to date, have investigated COVID-19 outcomes in patients with asthma.^13 16 18 19 21 25 33^ However, to date, the impact of the pandemic on asthma exacerbations is unclear. A recent study by Public Health England investigating causes of excess public deaths found a reduction in respiratory-related deaths (acute respiratory infections, chronic lower respiratory diseases and other respiratory diseases) during the pandemic when compared with deaths from the previous 5 years in England.^37^ Another recent study that aimed to derive and validate a risk prediction algorithm to estimate COVID-19 outcomes found that asthma does not increase risk of dying in either men or women, and only marginally increases the risk hospitalisation.^16^ These recent results support a reassuring viewpoint that patients with asthma are, perhaps, not as high risk as initially feared, and these studies align with our results that suggest an overall decrease in asthma exacerbations.

One possible explanation for a significant decrease only in exacerbation episodes that did not require hospital visit could be due to a change in careseeking behaviour. Some patients may have preferred exacerbating at home rather than reporting to primary care for fear of COVID-19 and a subset of those patients whose exacerbation did not resolve at home ended up visiting hospital for their asthma. It is also possible that some patients may have preferred self-referring themselves to A&E, as opposed to contacting primary care, so that they could be seen in person (in response to the pandemic, there was a shift from in-person to remote consultations in primary care across the UK^38^). However, by and large, there was an overall decrease in asthma exacerbations and while our study cannot definitively prove the reasons for this decrease, we believe that a combination of factors led to a reduction in asthma exacerbations. These factors include changing behaviour due to lockdown measures leading to reduction in air pollution, reduced circulation of respiratory viruses, improved self-management driven by patient concerns during the pandemic^39^ and shielding by a subset of patients (government-introduced UK-wide scheme where high-risk individuals are contacted and advised to follow stricter restrictions than the rest of the population^40^). We cannot, however, ascertain the role, if any, and extent of these factors in the current study. There is now a need to undertake further research (surveys of patient’s attitudes, and qualitative interviews with patients and primary care health professionals) to understand the social and disease-related mechanisms that can help explain our findings.

## Conclusion

Our study, the largest cohort study assessing the impact of COVID-19 on asthma to date, showed that there has been a significant reduction in asthma exacerbations (as recorded in primary care) during the pandemic and this reduction was observed in all age groups, both men and women and across most regions in England. This reduction in exacerbation rate was mainly seen in relation to exacerbations resolved within primary care without a need for a hospital visit. There is a need for further work to investigate the factors responsible for this decrease.
