# Correction: *Impact of COVID-19 national lockdown on asthma exacerbations: interrupted time-series analysis of English primary care data*

**DOI:** 10.1136/thoraxjnl-2020-216512corr1

**Published:** 2023-08-16

Shah SA, Quint JK, Nwaru BI, *et al*. Impact of COVID-19 national lockdown on asthma exacerbations: interrupted time-series analysis of English primary care data. Thorax 2021;76:860-866.

Author acknowledgements have been updated in the online HTML and PDF.

