# Supplementary material for: Impact of COVID-19 national lockdown on asthma exacerbations: interrupted time-series analysis of English primary care data
Source: Thorax. 2021 Mar 29;76(9):860–6. doi: 10.1136/thoraxjnl-2020-216512 (PMC8011425; doi:10.1136/thoraxjnl-2020-216512)
Supplement: Supplementary data [file thoraxjnl-2020-216512supp001.pdf]

Appendix 1 (Read Codes to Define Outcome)

Asthma exacerbation

Xa1hD","Xafdy","Xafdz","Xafdj","XE0YW","XM0s2","X101y","X1022","H333.","H3301","H3311","H33z0","H33z1

Asthma hospitalization or Accident & Emergency visit

"663d".,"8H2P.","663m."

Oral corticosteroids

"fe6..","fe3..","fe31.","fe32.","fe33.","fe36.","fe37.","fe3A.","fe3B.","fe3C.","fe3r.","fe3s.","fe3u.","fe4..","fe41.","fe42.","fe43.","fe44.","fe45.","fe4e.","fe4f.","fe4g.","fe4h.","fe5..","fe51.","fe52.","fe53.","fe5f.","fe5m.","fe5n.","fe5o.","fe5p.","fe61.","fe62.","fe64.","fe65.","fe66.","fe67.","fe68.","fe69.","fe6a.","fe6c.","fe6d.","fe6e.","fe6f.","fe6g.","fe6h.","fe6i.","fe6j.","fe6k.","fe6l.","fe6m.","fe6n., "fe6o.","fe6p.","fe6q.","fe6r.","fe6s.","fe6t.","fe6v.","fe6w.","fe6z.","fe7..","fe71.","fe72.","fe73.","fe74.","fe75.","fe76.","fe77.","fe78.","fe79.","fe7x.","fe7y.","fe7z.","x00yP","x01Mh","x01Na","x01Nb","fe11.","fe12.","fe1x.","fe1y.","fe21.","fe22.","fe23.","fe24.","fe25.","fe26.","x01MW"

Evidence of lower respiratory review

“XE1P1”,”XE2Nb”,”XE2b5”,”XM0rv”,”XM0s2”,”XM1Bs”,”XM1QV”,”XM1QX”,”XSCET”,”Xa0Y7”,”Xa0lY”,”Xa0lZ”,”Xa1hD”,”Xa35l”,”Xa7nL”,”Xa7nM”,”Xa7nN”,”Xa7nP”,”Xa7nT”,”Xa7nU”,”Xa8Hn”,”Xa97Z”,”Xa9zf”,”XaBE9”,”XaBM8”,”XaBfJ”,”XaDcD”,”XaDcV”,”XaDsa”,”XaDtB”,”XaDtP”,”XaDth”,”XaDtl”,”XaDvK”,”XaDvL”,”XaEFy”,”XaEFz”,”XaEIV”,”XaEIW”,”XaEIY”,”XaF6d”,”XaF6e”,”XaFrU”,”XaFrV”,”XaFrW”,”XaFrX”,”XaIIW”,”XaIIX”,”XaIIY”,”XaIIZ”,”XaIND”,”XaINZ”,”XaINa”,”XaINb”,”XaINc”,”XaINd”,”XaINf”,”XaINg”,”XaINh”,”XaIQ2”,”XaIQ3”,”XaIQ4”,”XaIQD”,”XaIQE”,”XaIQg”,”XaIR3”,”XaIUi”,”XaIUl”,”XaIUm”, ”XaIUn”,”XaIUo”,”XaIeq”,”XaIer”,”XaIet”,”XaIfK”,”XaIoE”,”XaIu5”,”XaIu6”,”XaIuG”,”XaIww”,”XaIxQ”, ”XaIxR”,”XaIxU”,”XaIxV”,”XaJ3K”,”XaJ9B”,”XaJ9C”,”XaJ9D”,”XaJ9E”,”XaJEl”,”XaJFG”,”XaKdk”,”XaLIm”,”XaLIn”,”XaLIr”,”XaLJO”,”XaLJS”,”XaLJT”,”XaLJU”,”XaLPE”,”XaMeu”,”XaN4a”,”XaNKw”,”XaObi”,”XaObj”,”XaObk”,”XaObl”,”XaObm”,”XaPpI”,”XaQHq”,”XaQig”,”XaQih”,”XaQmR”,”XaQmS”,”XaQmT”,”XaQmU”,”XaREU”,”XaRFi”,”XaRFj”,”XaRFk”,”XaRFl”,”XaVx3”,”XaX3n”,”XaXCa”,”XaXCb”,”XaXZm”,”XaXZp”,”XaXZs”,”XaXZu”,”XaXZx”,”XaXa0”,”XaXeg”,”XaY2V”,”XaYYt”,”XaYYu”,”XaYZB”,”XaYZh”,”XaYb8”,”XaYby”,”XaYja”,”XaYmL”,”XaYpC”,”XaYpF”,”XaZ1k”,”XaZ1l”,”XaZd1”,”XaZyl”,”XaZyt”,”XaZyu”,”XaZz1”,”Xaa7B”,”Xaa7C”,”Xaa7Q”,”Xaafd”,”XaahD”,”Xab2f”,”Xab4X”,”ZV19L”,”ZV725”,”ZV74B”,”ZV7B3”,”c74..”

Additional non-asthma specific codes for hospitalisation

“8HJJ.00”,”9b8D.00”,”8HX..00”,”8HX2.00”,”8Hd..00”,”8H16.00”,”8H2R.00”,”8H29.00”,”8H2D.00”,”8H2J.00”,”8H24.00”,”8H26.00”,”8H2H.00”,”8H2Z.00”,”8H21.00”,”8H2E.00”,”8H2N.00”,”8H27.00”,”8H2B.00”,”8H2K.00”,”8H28.00”,”8H25.00”,”8H2I.00”,”8H23.00”,”8H2L.00”,”8H2G.00”,”8H2M.00”,”8H2C.00”,”8H22.00”,”8H1..11”,”8H15.00”,”8H1Z.00”,”8H1..00”,”8H14.00”,”8H13.00”,”8H12.00”,”8H2A.00”,”8H2F.00”,”SP31100”,”663m.00”,”66Yd.00”,”9b8B.00”,”9498”,”8HG..11”,”9451”,”L398400”, ”8HG..00”,”ZLD2G11”,”ZLD2100”,”ZLD2H11”,”ZLD2H00”,”ZLD2111”,”ZLD2G00”,”ZLD2I11”,”ZLD2I00”,”ZLE1.00”,”ZLE5111”,”ZLE5100”,”ZLE1.11”,”ZLF2.00”,”ZLE5.00”,”ZLE5200”,”ZLG6.00”,”ZLG6100”,”ZLG6400”,”ZLG6500”,”8HE8.00”,”8HE..00”,”8HE2.00”,”8HN..00”,”7A10000”,”7A15000”,”7A12000”,”7A41400”,”7A47800”,”7A47200”,”7A47000”,”7A47600”,”7A47400”,”7A47C00”,”7A41000”,”7A41200”,”7A15300”,”7A41600”,”7A47B00”,”7A47300”,”7A47700”,”7A47100”,”7A47D00”,”8H23000”,”7A11000”,”7A11200”,”7A45300”,”7A13400”,”7A13000”,”7A45200”,”7A45000”,”7A40000”,”7A13300”,”7A45700”,”7A45D00”,”7A13100”,”66Ye.00”,”8H2P.00”,”7700.11”,”7700300”,”663d.00”,”7F13300”,”7700100”,”7700000”,”7700”,”7700z00”,”7700200”,”7A41211”,”8H2..00”,”8712”,”7M30000”,”7A13.

Shah SA, *et al. Thorax* 2021; 76:860–866. doi: 10.1136/thoraxjnl-2020-216512

11”,”7A45.15”,”7A45.00”,”7A45.14”,”7A13.00”,”7A13z00”,”7A13y00”,”8H2T.00”,”7700400”,”9239”,
”9b0K.00”,”945Z.00”,”945..00”,”13F8.11”,”9b0L.00”,”13F8.00”,”9R6..00”,”7936500”,”8HN1.00”,”8H
NA.00”,”8HNB.00”,”8HNC.00”,”8HND.00”,”8HNE.00”,”8HN2.00”,”8HN3.00”,”8HN4.00”,”8HN5.00”,”
8HN6.00”,”8HN7.00”,”8HN8.00”,”8HN9.00”,”8HN0.00”,”8HNZ.00”,”8CO..00”,”Z177800”,”8A8..00”,”
8Hb..00”,”8HM4.00”,”8HMB.00”,”8HM3.00”,”8HME.00”,”8HMJ.00”,”8HM8.00”,”8HM1.00”,”8HMG.
00”,”8HM7.00”,”8HMO.00”,”8HM5.00”,”8HM6.00”,”8HML.00”,”8HMN.00”,”8HMD.00”,”8HMH.00”,
”8HMQ.00”,”8HMK.00”,”8HMM.00”,”8HM2.00”,”8HMP.00”,”8HM9.00”,”8HMC.00”,”8HMA.00”,”8H
MR.00”,”8HM..00”,”8HMZ.00”,”8HMF.00”,”13F8100”,”13FS.00”,”66Yi.00”,”8H32.00”,”7701.11”,”8H
3E.00”,”8H3V.00”,”8H3T.00”,”8H3I.00”,”8H3O.00”,”8H39.00”,”8H3B.00”,”8H3M.00”,”8H31.00”,”8H
3..00”,”8H36.00”,”8H3J.00”,”8H3S.00”,”8H3C.00”,”8H3G.00”,”8H3U.00”,”8H3D.00”,”8H3A.00”,”8H3
N.00”,”8H38.00”,”8H3Q.00”,”8H3L.00”,”8H3R.00”,”8H3P.00”,”8H3H.00”,”8H37.00”,”8H3F.00”,”8H3
K.00”,”7A47.11”,”7A47.12”,”7A47.13”,”7A47.16”,”7A47.00”,”7A47z00”,”7A47y00”,”7A47.14”,”7A15
.00”,”7A47.15”,”8H3Z.00”,”7700y00”,”7259y00”,”7B07y00”,”7513y00”,”9b8C.00”,”949B.00”,”9495”
,”9144”,”9N19.11”,”7B07.00”,”7B07z00”,”T772.00”,”9b8A.00”,”8HLP.00”,”8HKP.00”,”8HJI.00”,”8HC
1.00”,”8H63.00”,”ZL51.11”,”8HC3.00”,”8H7a.00”,”8HD..00”,”8HC..00”,”8HCZ.00”,”ZL56.11”,”ZL51.0
0”,”ZL56100”,”ZL51.13”,”8HTF.00”,”ZL56.00”,”ZL56211”,”ZL56200”,”8HX1.00”,”9H4..00”,”9H5..00”,
”ZL96.11”,”ZL91.00”,”ZL96111”,”ZL96100”,”ZL91.12”,”ZL96211”,”ZL96200”,”ZL9GQ00”,”9N04.00”,”
9N1y300”,”9N19.00”,”9N1B.00”,”8HJ..00”,”8HJZ.00”,”8HF..12”,”7A13411”,”ZL16.11”,”ZL11.00”,”ZL1
6111”,”ZL16100”,”ZL11.12”,”ZL16.00”,”ZL16211”,”ZL16200”,”8HX0.00”,”8Ha..00”,”7A11211”,”ZV29
300”,”ZV29211”,”ZV29200”

<u>Specific asthma codes previously validated to identify asthma patients in primary care</u>

“H33..”,”663..”,”H333.”,”H33z1”,”H33z0”,”H33..”,”H330.”,”663V1”,”663V3”,”663V0”,”H331.”,”H33z.
”,”H33zz”,”H33z0”,”H331.”,”H3300”,”H3120”,”173A.”,”H3301”,”8H2P.”,”H330.”,”663P.”,”663U.”,”6
63N.”,”H330.”,”H33z1”,”9OJA.”,”663y.”,”66Y5.”,”66Y9.”,”66YJ.”,”8B3j.”,”663j.”,”1O2..”,”H33z2”,”66
3V.”,”663V2”,”663N2”,”66YK.”,”H3300”,”H330.”,”H33zz”,”8795”,”8794”,”66YA.”,”8796”,”H3311”,”6
6YQ.”,”663p.”,”663n.”,”9OJA.”,”8798”,”8797”,”H33zz”,”173c.”,”663d.”,”8791”,”663u.”,”663e.”,”8CR
0.”,”663s.”,”663v.”,”663f.”,”663e1”,”663e0”,”H3301”,”H3310”,”66YR.”,”663N0”,”66YP.”,”663t.”,”66
3O0”,”663w.”,”663x.”,”663N1”,”H35y7”,”663r.”,”H334.”,”1780”,”66YC.”,”663q.”,”H331z”,”H330z”,”
9OJ1.”,”663m.”,”H47y0”,”H3311”,”173d.”,”H35y6”,”388t.”,”38DL.”,”8CMA0”,”679J0”,”38DT.”,”9NN
X.”,”679J1”,”66Yp.”,”38DV.”,”1787”,”1781”,”66Yr.”,”66Yq.”,”1789”,”663P0”,”178B.”,”663P1”,”1783
”,”679J2”,”1786”,”66Ys.”,”388t0”,”1788”,”178A.”,”1785”,”1784”,”1782”,”663P2”,”661N1”,”661M1”
,”H335.”,”66Yu.”
