# Supplementary material for: Impact of COVID-19 national lockdown on asthma exacerbations: interrupted time-series analysis of English primary care data
Source: Thorax. 2021 Mar 29;76(9):860–6. doi: 10.1136/thoraxjnl-2020-216512 (PMC8011425; doi:10.1136/thoraxjnl-2020-216512)
Supplement: Supplementary data [file thoraxjnl-2020-216512supp002.pdf]

Appendix 2

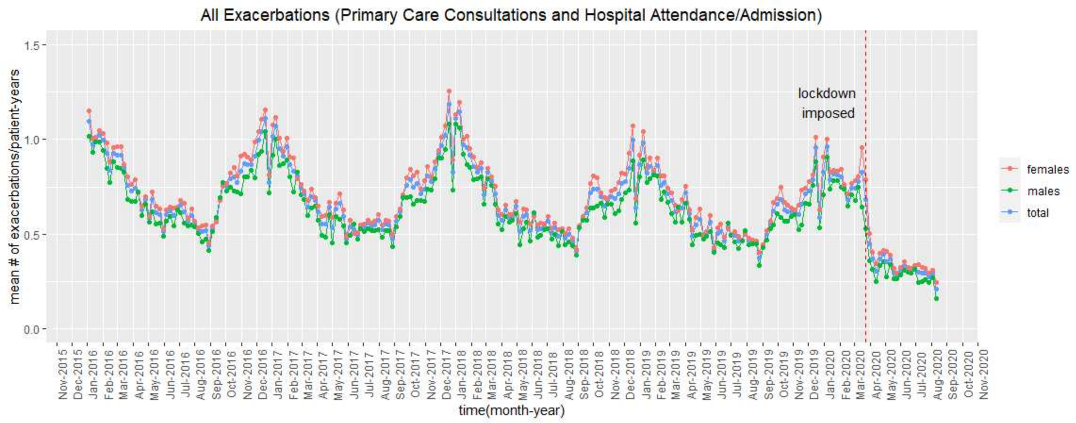

Figure S1: Mean exacerbation rate for every week, from January 2016 - August 2020 across England stratified by gender

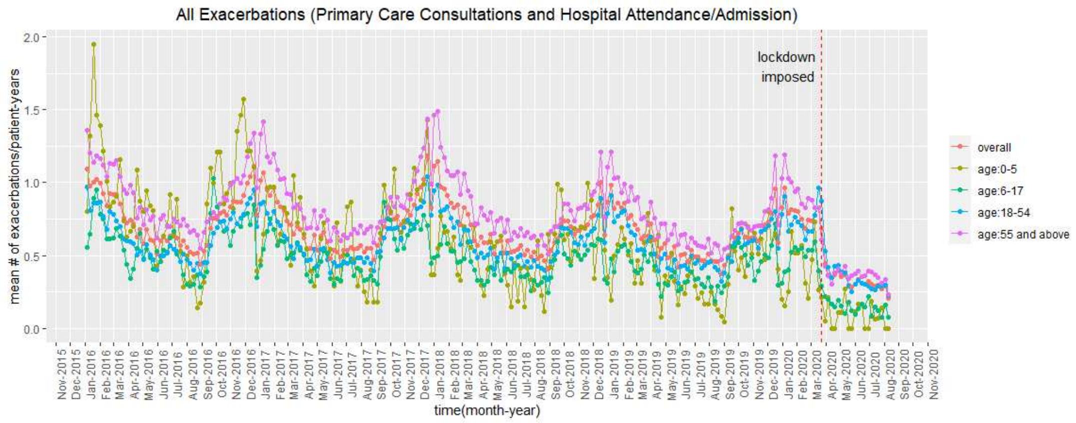

Figure S2: Mean exacerbation rate for every week, from January 2016 - August 2020 across England stratified by age group

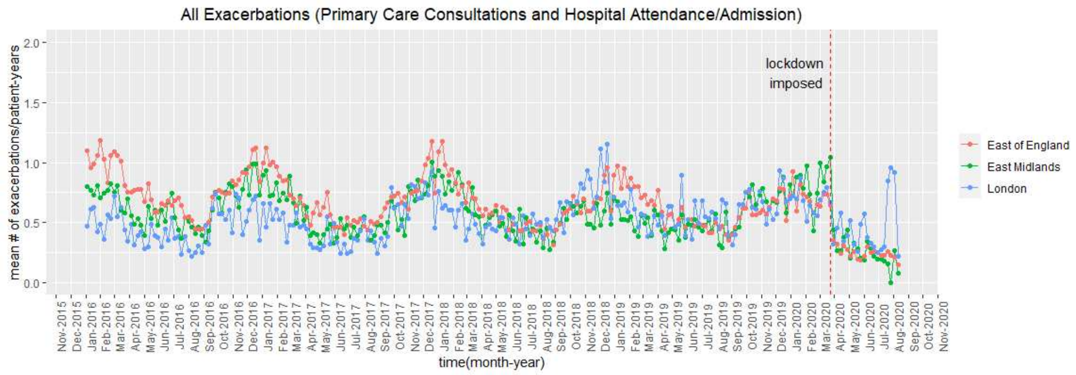

Figure S3: Mean exacerbation rate for every week, from January 2016 - August 2020 across England stratified by region (East of England, East Midlands, London)

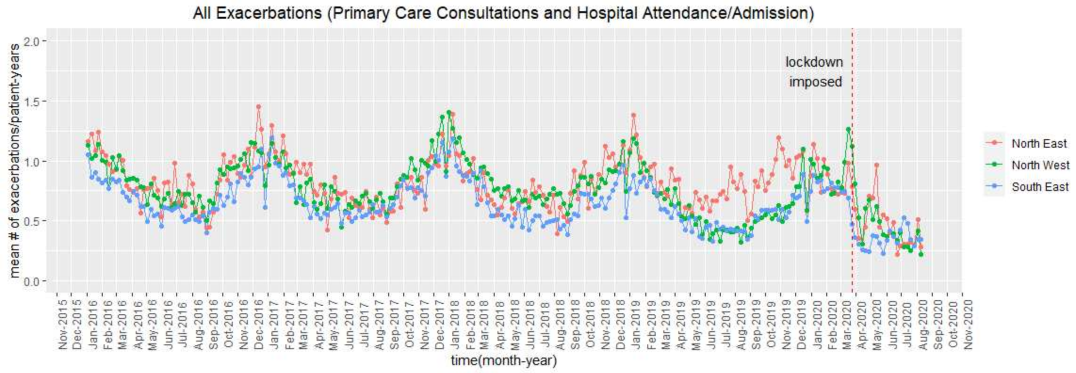

Figure S4: Mean exacerbation rate for every week, from January 2016 - August 2020 across England stratified by region (North East, North West, South East)

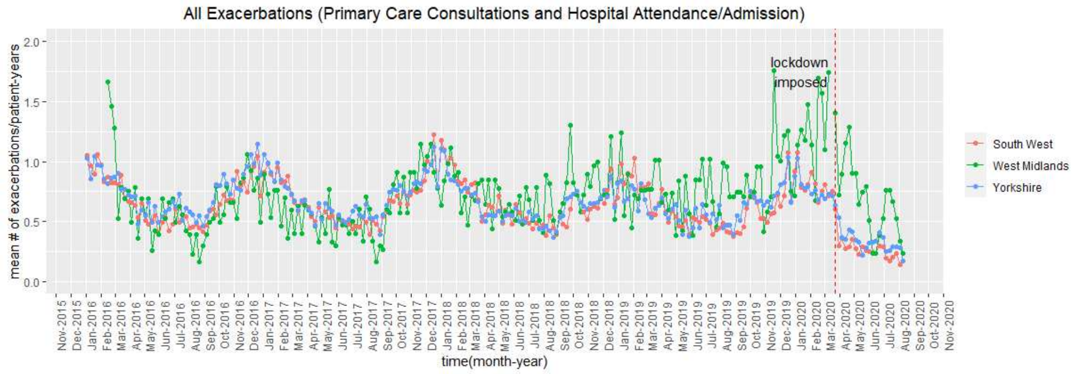

Figure S5: Mean exacerbation rate for every week, from January 2016 - August 2020 across England stratified by region (South West, West Midlands, Yorkshire)
